# The Medical Practitioners’ and Students’ Library, No. 5; The Druggists' General Receipt Book

**Published:** 1850-05

**Authors:** 


Medical Practitioners' and Students' Library, No. 5. *The American Medical Formulary: based upon the United States and British Pharmacopœias. Including also numerous standard formulæ, derived from American and European authorities. Together with the medical properties and uses of Medicines; Poisons, their antidotes, tests, &c.; designed for the medical and pharmaceutical student.* By John J. Reese, Lecturer on Materia Medica and Therapeutics in the Philadelphia Medical Institute, &c. Philadelphia. Lindsay & Blakiston. 1850.

*The Druggists' General Receipt Book: containing numerous recipes for patent and proprietary medicines, Druggists' nostrums, etc.; Factitious Mineral Waters, and powders for preparing them; with a Veterinary Formulary, and table of veterinary Materia Medica; also recipes for perfumery and*

*cosmetics ; beverages, dietetic articles and condiments ; trade chemicals, miscellaneous compounds used in the arts, domestic economy, &c.; with useful tables and memoranda.* By Henry Beasley. Philadelphia. Lindsay & Blakiston. 1850.

In the last number of the Examiner our views in relation to Formularies were freely expressed; it needs not, therefore, that a repetition of them should be presented to the reader at this time in connection with the works whose titles head this article. To the author of the first named we gladly assign an honored place among those who are engaged in facilitating the onward progress of the art of medicine. Although we have our doubts, as was before expressed, whether the means employed in the present instance are the best that could be adopted to advance the end. There is, nevertheless, something in the name, The *American* Medical Formulary, that knocks loudly at the door of our sympathies, and the hand of welcome is extended almost without inquiry. The term *American,* the author thinks, "may, without impropriety, be assigned to it, since, throughout the work, in the description of the various medicinal substances and pharmaceutical preparations, the precedence has uniformly been given to those recognized by our national Pharmacopœia." We regret that the publication was not delayed a little until the revision of the U. S. Pharmacopœia, just to take place, had been completed, and the result of the deliberations of that Convention made known.

In the arrangement of the work the alphabetical order has been adopted, for the two-fold reason of easy reference, and the avoidance of any classification of medicines, which is always more or less arbitrary. Under this arrangement, every article that has a place in the Pharmacopœia of the United States and in those of Great Britain, has been described; together with a condensed notice of its medical properties, uses, and mode of preparation. To these are added some of the best and most valuable of the numerous published prescriptions, both of hospitals and private practice, with, in nearly every case, the name of the author.

In the Appendix will be found a list of some of the more common and useful dietetic preparations; a brief description of poisons, with reference to their treatment, antidotes, and tests; a table of signs and abbreviations; one of proportional doses; one of weights and measures; together with a table of the most celebrated mineral waters of the United States and Europe; and one, of the doses of the most important medicines, all of which cannot fail to be of interest to the reader.

The author has manifested much care and research in the preparation of the volume before us, and has presented us with a work, in general, of great accuracy, which cannot fail to be useful for reference, both to the practitioner and student, if used with proper caution, and not relied upon to the exclusion of his own powers of discrimination and judgment. But, as we have before observed, the danger of all formularies is, that the young practitioner seeing a formula prepared for some special malady, and employed by some well known authority, adopts it unhesitatingly, and thus runs the great risk of becoming a mere routinist. To this objection, however, the American Formulary is less open than any other that we have seen, and we, therefore, with more confidence commend it to the profession.

The work itself is one of a series now in course of preparation, and of which five, including the present, have already appeared. The publishers are entitled to the thanks of the profession, for the desire they have manifested to supply good and sound material and at so cheap a rate, and we hope that they may meet with the success their enterprise so richly deserves. Its mechanical execution is excellent.

The second work is a reprint from the English press, and intended for the use of the druggist, who will find in it a large supply of matter of value to him in his profession. To the country druggist and storekeeper it will prove particularly valuable. As we are without experience, however, in veterinary practice, and in the preparation of perfumery, cosmetics, &c., we can give no opinion as to the merits of the different formulæ contained in it. The title page presents, in itself, an analysis of its contents, which, it will be seen, covers a large extent of surface. In the division of *Trade Chemicals* are contained those compounds which are employed for other purposes than those of medicine, among which we have found much that will prove useful to the practical manipulator in almost every department of art, inasmuch as it supplies him with information whereby much expense may be saved in the preparation of his materials.

